# Expression and cellular localization of microRNA-29b and RAX, an activator of the RNA-dependent protein kinase (PKR), in the retina of streptozotocin-induced diabetic rats

**Published:** 2011-08-18

**Authors:** Viviane A. O. Silva, Anna Polesskaya, Thaís A. Sousa, Vani M. A. Corrêa, Nayara Delgado André, Rosana I. Reis, Isis C. Kettelhut, Annick Harel-Bellan, Fernando L. De Lucca

**Affiliations:** 1 Department of Biochemistry and Immunology, School of Medicine University of São Paulo, Ribeirão Preto, São Paulo, Brazil; 2 University Paris-Sud CNRS, FRE 2944, University Paris-Sud, Villejuif, France

## Abstract

**Purpose:**

The apoptosis of retinal neurons plays a critical role in the pathogenesis of diabetic retinopathy (DR), but the molecular mechanisms underlying this phenomenon remain unclear. The purpose of this study was to investigate the cellular localization and the expression of microRNA-29b (*miR-29b*) and its potential target PKR associated protein X (RAX), an activator of the pro-apoptotic RNA-dependent protein kinase (PKR) signaling pathway, in the retina of normal and diabetic rats.

**Methods:**

Retinas were obtained from normal and diabetic rats within 35 days after streptozotocin (STZ) injection. In silico analysis indicated that RAX is a potential target of *miR-29b*. The cellular localization of *miR-29b* and RAX was assessed by in situ hybridization and immunofluorescence, respectively. The expression levels of *miR-29b* and RAX mRNA were evaluated by quantitative reverse transcription PCR (qRT–PCR), and the expression of RAX protein was evaluated by western blot. A luciferase reporter assay and inhibition of endogenous RAX were performed to confirm whether RAX is a direct target of *miR-29b* as predicted by the in silico analysis.

**Results:**

We found that *miR-29b* and RAX are localized in the retinal ganglion cells (RGCs) and the cells of the inner nuclear layer (INL) of the retinas from normal and diabetic rats. Thus, the expression of *miR-29b* and RAX, as assessed in the retina by quantitative RT–PCR, reflects their expression in the RGCs and the cells of the INL. We also revealed that RAX protein is upregulated (more than twofold) at 3, 6, 16, and 22 days and downregulated (70%) at 35 days, whereas *miR-29b* is upregulated (more than threefold) at 28 and 35 days after STZ injection. We did not confirm the computational prediction that RAX is a direct target of *miR-29b*.

**Conclusions:**

Our results suggest that RAX expression may be indirectly regulated by *miR-29b,* and the upregulation of this miRNA at the early stage of STZ-induced diabetes may have a protective effect against the apoptosis of RGCs and cells of the INL by the pro-apoptotic RNA-dependent protein kinase (PKR) signaling pathway.

## Introduction

The discovery of microRNA (miRNA) added a new layer of complexity to the regulation of gene expression [1]. MiRNAs are endogenous, noncoding RNAs (19–25 nucleotides in length) that regulate gene expression via binding to specific sites at the 3′-untranslated region (3′-UTR) of the target mRNAs, thereby causing translational repression or mRNA degradation. Recent computational predictions indicate that miRNAs may regulate approximately 30% of human protein-coding genes [2]. In mammals, the functions of specific miRNAs have been described in important cellular processes, such as cell-cycle regulation [3] and hematopoietic [4] and adipocyte [5] differentiation. The abnormal expression of miRNAs has recently been associated with several diseases. However, little is known about the role played by miRNAs in ocular diseases [6,7].

Diabetic retinopathy (DR) is one of the most common complications of diabetes, and nearly all people with type 1 and more than half with type 2 diabetes develop retinopathy [8]. Accumulating evidence indicates that apoptosis of the retinal neurons precedes the microvascular alterations in the retina of diabetic patients and streptozotocin (STZ)-induced diabetic rats [9,10]. It has also been demonstrated that apoptosis of the retinal neurons plays a critical role in the pathogenesis of DR [11], but the molecular mechanisms underlying this phenomenon are currently unclear.

Recent findings indicated that microRNA-29b (*miR-29b*) is involved in apoptosis [12,13]. Based on the finding that the *miR-29* family is expressed in the rat retina [14] and that one miRNA has several targets, we hypothesize that *miR-29b* could regulate the genes in the pro-apoptotic pathways that are involved in the apoptosis of the retinal neurons of STZ-induced diabetic rats. The activation of the RNA-dependent protein kinase (PKR) is reported to be involved in the cell death of the retinal ganglion cells treated with tunicamycin [15]. These authors showed that the activation of PKR is due to the endoplasmic reticulum (ER) stress induced by tunicamycin. Moreover, it was demonstrated that ER stress has a role in the early stage of diabetic retinopathy [16]. Interestingly, the in silico analysis revealed that one of the potential targets of *miR-29b* is RAX (PK*R-a*ssociated protein *X*), the only known physiologic activator of PKR. It has been suggested that RAX may be a direct stress-sensitive activator of PKR and could induce apoptosis by activating the PKR-signaling pathway under stress conditions [17]. In this context, it is possible that the sustained hyperglycemic state and consequent ER stress observed in diabetes could induce the expression of RAX in the retinal neurons of diabetic animals.

In the present study, we used STZ-induced diabetic rats because this experimental model of diabetes displays many of the morphological and functional changes in the retinal neurons that are evident in human DR [18]. We investigated the cellular localization and expression of *miR-29b* and RAX in the retina of normal and diabetic rats to elucidate their possible involvement in the apoptosis of retinal neurons.

## Methods

### Animals and treatment with streptozotocin

Male Wistar rats weighing 130 to 150 g were housed in suspended wire-bottom cages in a room kept at 25±2 °C with a 12:12 h light–dark cycle and were provided with food and water ad libitum. The animals were randomly divided into groups of eight. For the induction diabetes, STZ (Sigma Chemical Co., St. Louis, MO) was dissolved in 0.01 M citrate buffer, pH 4.5, and was injected within 5 min of its preparation. The rats were fasted overnight, anesthetized with isoflurane, and injected with STZ in the jugular vein at a dose of 45 mg/kg bodyweight. The control rats were injected with the citrate buffer [19]. Blood glucose levels were measured by the colorimetric oxidase glucose method (Labtest, Lagoa Santa, Brazil) 24 h after the injection of STZ or citrate buffer. Only animals with blood glucose values ≥400 mg/dl were used. All experiments were performed between 8:00AM and 10:00 AM The STZ-injected and control rats were sacrificed by rapid cervical dislocation at the indicated time points after treatment. The retinas were dissected and used either for the analysis of RAX mRNA and *miR-29* expression and protein analysis or processed for in situ hybridization and immunofluorescence. The care and treatment of the animals received prior institutional approval (protocol 012/2008) from the Ethical Commission of Ethics in Animal Research of the School of Medicine at the University of São Paulo, Ribeirão Preto, SP, Brazil.

### Tissue fixation

For in situ hybridization and immunofluorescence, enucleated eyes were first immersion fixed in 4% buffered paraformaldehyde prepared in 0.2 M phosphate buffer (pH 7.3) at 4 °C for 24 h, transferred to cassettes, and subsequently treated with 70% ethanol for 2 h, 99% ethanol for 1 h, absolute ethanol for 30 min, xylene at room temperature for 1 h, xylene at 37 °C for 10 min, then in a paraffin bath at 56 °C for 1 h. The fixed samples were subjected to vacuum pressure for 30 min and then embedded in paraffin. The paraffin blocks were cut into 5-μm serial sections that were placed on Superfrost^TM^ (Cole-Parmer, Vernon Hills, IL) slides. Cellular integrity was assessed by histological staining using Harris hematoxylin and eosin-phloxine. For this, the sections were adhered to the slides in an oven at 60 °C, deparaffinized in xylene, and rehydrated through graded ethanol washes (100%, 95%, 80%, and 70%) for 10 min each followed by a wash in distilled water for 5 min. Subsequently, the sections were stained with hematoxylin for 1 min, washed in distilled water, and stained with eosin for 2 min. The slides were dehydrated and mounted in Permount (Fisher Scientific, Pittsburgh, PA). Other sections of each sample were processed for immunofluorescence and in situ hybridization.

### Immunofluorescence

For immunostaining, the slides were incubated in 0.1 M glycine (dissolved in PBS: 0.01 M phosphate-buffered saline, pH 7.4) for 30 min and blocked for 1 h in 1% goat serum, 2% BSA (BSA), and 0.05% Triton X-100 in PBS at room temperature in a humidity chamber. The slides were incubated in the primary anti-PACT (PKR activator) goat polyclonal antibody (Santa Cruz Biotechnology, Santa Cruz, CA), diluted 1:50 in blocking buffer under the same conditions for 3 h. After three 5-min washes in PBS, slides were incubated in a 1:2,000 Alexa Fluor 594 rabbit antigoat fluorescent secondary antibody solution (Molecular Probes, Invitrogen) in a humidified chamber for 1 h at room temperature in the dark. The slides were washed ten times in PBS, mounted with Vectashield mounting medium with with the 4',6-diamidino-2-phenylindole (DAPI; Vector Laboratories, Burlingame, CA), and coverslips were applied. The slides were viewed with a Leica DM5500B fluorescent microscope (Leica Microsystems, Bannockbur, IL). The images were captured with the Leica DFC *340 FX* software (Leica Microsystems) and processed using using Adobe PhotoShop software (Adobe Systems Inc., San Jose, CA). The specificity of antibody staining was confirmed by incubating the adjacent sections in the absence of the primary antibody.

### In situ hybridization

To perform in situ hybridization of *miR-29b*, a locked nucleic acid (LNA)-modified, digoxigenin (DIG)-labeled probe was generated by Exiqon (Vedbaek, Denmark). Such LNA probes have previously been shown to provide exceptional miRNA hybridization specificity that is sensitive to 1 or 2 nucleotide mismatches [20]. We optimized the miRNA in situ hybridization protocol described by Ryan et al. [21]. In this protocol, deparaffinized sections were treated for 15 min with 0.1% H_2_O_2_ to block endogenous peroxidase activity and treated for 5 min with proteinase K (10 μg/ml) followed by 30 s in 0.2% glycine. The sections were refixed in 4% paraformaldehyde for 10 min, washed twice in PBS, and prehybridized for 2 h in the hybridization buffer (50% formamide, 5×300 mM NaCl, 30 mM sodium citrate, pH 7.0, 0.4% Tween, 9.2 mM citric acid [pH 6], 50 μg/ml heparin, 500 μg/ml yeast RNA). The tissues were hybridized overnight at room temperature in the presence of 20 nM of the DIG-labeled probe. The slides were washed twice in 2× SSC for 5 min, followed by three 30-min washes in 50% formamide plus 2× SSC at 37 °C. After five washes in 0.1% Triton X-100 in PBS, the slides were incubated in blocking solution (2% sheep serum, 2 mg/ml BSA in PBST: 0.1M PBS, 0.5% Tween-20, pH 7.4) followed by an overnight incubation at 4 °C in a 1:1,000 of anti-digoxigenin monoclonal antibody conjugated with biotin (Abcam, Cambridge, UK). The slides were washed seven times for 5 min in PBS. Immunological detection was performed with the TSA (Tyramide Signal Amplification) Biotin System (Perkin Elmer, Boston, MA) for 10 min at room temperature. The specificity of the antibody staining was confirmed by incubating adjacent sections in the absence of the *miR-29b* antibody. As a positive control, a U6 probe generated by Exiqon was substituted for the *miR-29b* probe.

### RNA isolation

Total RNA was extracted from the retinas of normal and diabetic rats using Trizol-LS Reagent (Invitrogen, Carlsbad, CA) followed by isopropanol precipitation. RNA concentration was assessed using a biophotometer (Eppendorf, Hamburg, Germany), and a ratio of 2.0 for sample absorbance at 260/280 nm was considered acceptable.

### Analysis of microRNA-29b expression by quantitative reverse transcription PCR

The expression analysis of mature *miR-29b* was performed with quantitative reverse transcription PCR (qRT–PCR) for the retina samples at various intervals after STZ treatment and for the control retinas. Briefly, 2.5 µl of total RNA (10 ng) was supplemented with the RT primer mix with the RT primer (TaqMan MicroRNAs Reverse Transcription Kit, Applied Biosystems, Foster City, CA). For β-actin assays, RT reactions were performed using 500 ng of RNA and random hexamers (Applied Biosystems). All reactions were incubated in the Thermo Hybaid PCR Express (Middlesex, UK) at 42 °C for 1 h. After the RT reaction, the cDNA products were diluted to 1:4. In a 10-μl PCR reaction, 4.0 µl of the diluted cDNA was added to 5.0 μl of 2× PCR master mix (TaqMan Universal PCR master mix; Applied Biosystems) and 1.0 μl of the miR-29b primers and TaqMan probe mix; Applied Biosystems). The same procedure was used for the β-actin assays except for the use of 4.5 µl of the diluted cDNA and 1.5 μl of the human β-actin primers and probe mix (predeveloped TaqMan assay; Applied Biosystems). The reactions were incubated in a 96-well optical plate at 95 °C for 10 min followed by 40 cycles of 95 °C for 15 s and 60 °C for 10 min, using an Applied Biosystems 7500 Sequence Detection system. The cycle threshold (ΔCt) values were calculated, and the data were normalized to the housekeeping gene β-actin (*ACTB*) [22]. Real-time PCR was performed in triplicate in three independent experiments, and the results were expressed as means±standard error of the mean (SEM).

### Prediction of microRNA-29b targets

We used three algorithms to computationally predict potential targets of miR-29b. The algorithms included miRanda, TargetScan 5.0, and FindTar. The computationally predicted miRNA–target pairs were downloaded to a local database. We selected the best predicted targets of miR-29b by comparing the results from the three target prediction databases [23].

### Analysis of the PKR activator RAX mRNA expression by quantitative reverse transcription PCR

Standard SYBR Green qRT–PCR was performed to detect the *RAX* transcript levels in the total cellular RNA from the retinas of the hyperglycemic and the normoglycemic rats. After RNA isolation, 1.2 µg of RNA from the individual samples was reverse transcribed using the Superscript III Reverse Transcriptase Kit (Invitrogen). The quantitative PCR (qPCR) assays were performed using an Applied Biosystems 7500 Sequence Detection system. The thermal cycling conditions for PCR were as follows: 95 °C for 3 min and 40 cycles of amplification comprising 95 °C for 12 s, 60 °C for 30 s, and 72 °C for 30 s. The reaction mixture consisted of 2 µl cDNA, SYBR Green PCR Master Mix (Applied Biosystems), and primers to *RAX* (sense 5′-AGC GGG ACC TTC AGT TTG G-3′ and antisense 5′-CTT GGT CTT CGT GCC GTA CTC-3′). These primers were specific for the published *RAX* cDNA sequence (GenBank NM_001024780.1), designed using Primer Express version 2 software (Applied Biosystems), and optimized for use at a final concentration of 2.5 µM. *ACTB* mRNA was analyzed in the same run using specific primers (sense 5′-TGG AAT CCT GTG GCA TCC ATG AAA C-3′ and antisense 5′-TAA AAC GCA GCT CAG TAA CAG TCC G-3′) as recommended by the manufacturer (Applied Biosystems). The Ct values were calculated, and the data were normalized to the housekeeping gene mRNA levels (*ACTB*). qPCR was performed in triplicate in three independent experiments with ±SEM reported. A linear standard curve analysis for each primer pair at a maximum of a 10^−6^ dilution of the control-pooled cDNA and a primer amplification efficiency (*E*) greater than 0.96 was obtained for all the PCR experiments. The heat dissociation of the amplified DNA detected a single peak in all cases, indicating that a single specific PCR product had been synthesized. This was confirmed by electrophoresis of the PCR products in which a single band of the expected molecular weight was observed.

### Western blot analysis

The retinas were lysed directly on ice in 200 µl of lysis buffer consisting of 20 mM Tris-HCl (pH 7.6), 50 mM KCl, 400 mM NaCl, 1 mM EDTA, 0.2 mM phenylmethylsulfonyl fluoride, 2 μg/ml aprotinin, 2 μg/ml leupeptin, 1 mM dithiothreitol, 1% Triton X-100, and 20% glycerol. The reagents used to prepare lysis buffer were purchased from Sigma Chemical Co., St. Louis, MO. The lysates were centrifuged at 10,000 ×g for 20 min, and the supernatant was stored at −70 °C. The soluble proteins were quantitated following the Lowry method [24]. Total cellular proteins (30 μg) were separated by electrophoresis through a 10% sodium dodecyl sulfate PAGE (SDS–PAGE)-resolving gel with an SDS–PAGE stacking gel. After electrophoresis, the proteins were transferred onto a Hybond-C-supported nitrocellulose membrane (Amersham Biosciences, Little Chalfont, UK) by semidry electroblotting. The membranes were blocked with 3% BSA in 100 mM Tris–HCl, pH 7.5, 100 mM NaCl, 0.1% Tween-20 (TBST) for 1 h and incubated in a 1:300 dilution anti-PACT goat polyclonal antibody (Santa Cruz Biotechnology) in blocking buffer for 1 h. After washing the membranes in TBST for 20 min, 1:2,000 dilution rabbit antigoat immunoglobulin secondary antibody conjugated to horseradish peroxidase (Amersham Biosciences) in TBST was added, and the membranes were incubated at room temperature for 60 min. After washing the membranes for 1 h min in diluted in TBST, Enhanced Chemiluminescence (ECL) Detection Reagents (Amersham Biosciences, Little Chalfont, UK) were added to the membranes for 2 min, and the membranes were developed on Hyperfilm (GE Healthcare, UK). The western blots were repeated three times; qualitatively similar results were obtained each time. Equal loading and transfer were ensured by reprobing the membranes for β-actin. The films were documented and the bands were analyzed by densitometry using ImageJ software. The bar graphs were plotted using GraphPad Prism 4.02 software (GraphPad, San Diego, CA).

### Luciferase reporter assay

The reporter plasmid containing the *miR-29b* complementary sequence was constructed with a fragment of the 3′UTR of RAX mRNA containing the predicted *miR-29b* binding site. This fragment was amplified by RT–PCR using primers (sense 5′-GCT GAG TGT GGC ATC CAT TT-3′ and antisense 5′-CCA CTT CAC AAA GCT TTG CAC-3′) that produced a 141-bp amplicon spanning nucleotides 189–330 and containing one potential target site for *miR-29b.* The amplicon was cloned in the pCR® 2.1 TOPO vector (Invitrogen) to incorporate the SacI–XbaI restriction enzyme sites. After the appropriate digestion and purification (QIAquick Gel Extraction and PCR Purification Kit; Qiagen), the product was subcloned into the XbaI site of site of pISO [25,26], downstream of the firefly luciferase-coding sequence, to generate the pISORAX plasmid. The plasmid was purified (Plasmid Purification Mini/Midi Kits; Qiagen), and successful cloning was confirmed by DNA sequencing. For transfection, human embryonic kidney cells (HEK293) were seeded in complete Dulbecco’s minimal essential medium (DMEM; Gibco-Invitrogen, Paisley, UK) supplemented with 15% fetal bovine serum (Dominique Dutscher, Brumath, France) in 24-well plates at a density of 5×10^4^ cells per well. After 24 h, the cells were transfected with Lipofectamine 2000 reagent (Invitrogen), 100 ng of the pISO reporter construct (pISO-REPORT-3′-UTR/*RAX* or pISO-REPORT empty), 20 ng of the *Renilla* luciferase control vector, pRL-TK (Promega, Lyon, France); and either a synthetic miR-29b mimic (Dharmacon, Denmark) or a short double-stranded RNA (dsRNA; Dharmacon), as a negative control at two doses (50 nM or 75 nM). The sequence (5′-GAT AGC AAT GAC GAA TGC GTA-3′) of the dsRNA control is not complementary to the 3′-UTR of *RAX* mRNA. The efficiency of transfection was monitored by reporter activity, which was measured using the dual luciferase assay kit (Promega) and a luminometer (Lecteur Microplaque-Mithras LB940). We measured firefly luciferase activity 24 and 48 h after transfection and normalized the results to the *Renilla* luciferase activity. We performed at least three independent experiments for each assay, and the results were expressed as the mean±SEM.

### Analysis of protein and mRNA expression levels of RAX, an activator of PKR, in the presence of a microRNA-29b mimic

To confirm the results of the luciferase reporter assay, CJ4 rat fibrosarcoma cells were seeded in six-well plates at a density of 1×10^6^ cells/well in DMEM supplemented with 15% fetal bovine serum. After 24 h, when they reached a confluence of 80%–85%, the cells were transfected at two doses (50 nM and 75 nM) with either the synthetic *miR-29b* mimic or the dsRNA negative control (described above) and 30 µl of Lipofectamine (Invitrogen, Carlsbad, CA) 2000. The cells were lysed at 24 and 48 h after transfection. Protein expression levels and RAX mRNA expression levels were assessed by western blot analysis and qRT–PCR, using the standard procedures already mentioned. We performed at least three independent experiments for each assay, and the results were expressed as the mean±SEM.

### Statistical analysis

Data are presented as mean±SEM. Differences between the control and the treated retinas were assessed with the paired Student *t* test, and p<0.05 was considered statistically significant. Analysis of the data was performed with GraphPad Prism 4.0 software (GraphPad Software, Inc., San Diego, CA) and Microsoft Excel.

## Results

### Cellular localization of microRNA-29b in the retina

**Figure 1:**
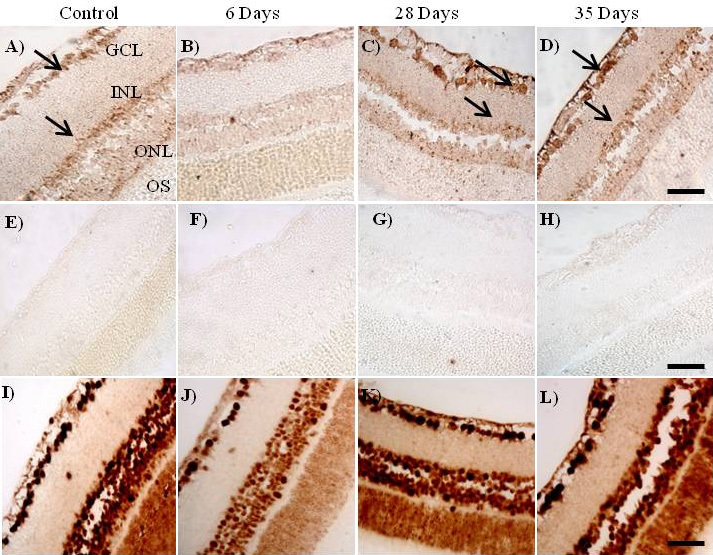
The cellular localization of microRNA-29b in the retina of streptozotocin (STZ)-induced diabetic rats by in situ hybridization. The *miR-29b* signal was weak in the retina of diabetic rats at 6 days (**B**), but strong at 28 days (**C**) and 35 days (**D**) after STZ injection when compared to normal rats (**A**). The negative controls (**E**, **F**, **G,** and **H**) were obtained with sections incubated in the absence of anti-digoxigenin (DIG)-labeled probe. The positive control sections (U6 RNA) of the normal and diabetic rat retinas at 6, 28, and 35 days are **I**, **J**, **K**, and **L**, respectively. Arrows indicate that *miR-29b* was expressed in the ganglion cell layer and the inner nuclear layer of the retinas. Original magnification was 400×. The scale bar represents 50 μm. Abbreviations are as follows: GCL represents ganglion cell layer; INL represents inner nuclear layer; ONL represents outer nuclear layer; OS represents photoreceptor outer segments. The results shown in this figure are representative of three replicates.

We used in situ hybridization to determine the cellular localization of *miR-29b* in the retina of diabetic rats. The cellular localization of *miR-29b* is shown in the control rat retinas (Figure 1A) and the diabetic rat retinas on days 6 (Figure 1B), 28 (Figure 1C), and 35 (Figure 1D) post-STZ injection. The *miR-29b* signal was weak at 6 days but strong at 28 and 35 days. The *miR-29b* staining was observed in the cytoplasm, and there was no change in the cellular localization of *miR-29b* in the retina of normal and diabetic rats.

### Expression of microRNA-29b in retina

**Figure 2:**
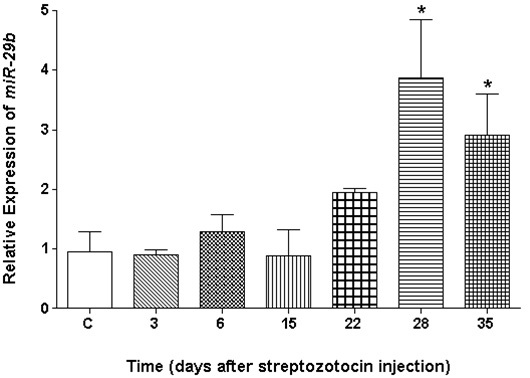
The expression of *microR-29b* in the retina of normal and streptozotocin-induced diabetic rats. The mature *miR-29b* expression was evaluated by Taqman real-time qRT–PCR in the retinas of normal and diabetic rats. The total RNA of the retina was reverse transcribed using *miR-29b* and β-actin-specific primers, and qPCR experiments were performed as described in the methods. The expression of the mature *miR-29b* was normalized to β-actin. Rats were used in groups of three. Data are means±standard error of the mean from three independent experiments, and each sample was run in six replicates. The paired Student *t* test was performed to ascertain whether there is statistical significance between the *miR-29b* expression levels in the retinas of diabetic rats and nondiabetic rats. The asterisk (*) indicates statistical significance at p<0.05.

The distribution of *miR-29b* was strongest and primarily seen in the ganglion cell layer (GCL). Very little signal was detected in the inner nuclear layer (INL), and little if any expression was detected in the outer nuclear layer (Figure 1C,D). The analysis of the expression of *miR-29b* in the retina by qRT–PCR reflects the expression seen in the RGCs and cells of the INL, as indicated in Figure 1C. We observed that *miR-29b* is upregulated (>3 fold) at 28 (p=0.01) and 35 (p=0.05) days after injection of STZ (Figure 2). The expression profiles of *miR-29a* and *miR-29c* were similar to that found for *miR-29b* (data not shown).

### Computational prediction of potential microR-29b targets

**Figure 3:**
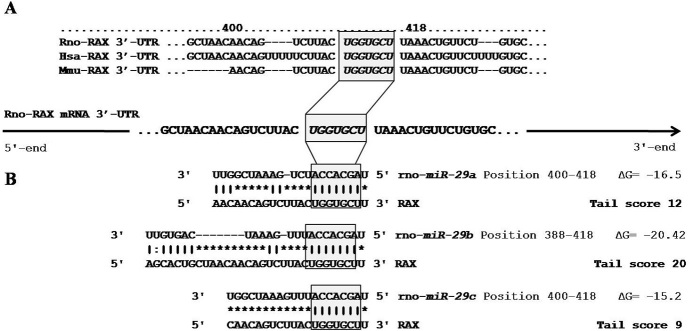
The analysis of potential *microR-29b* binding sites within the 3′-UTR of PKR associated protein X (RAX) mRNA. The bioinformatic algorithms TargetScan, miRanda, and FindTar were used to predict *miR-29* binding sites in the 3′-UTR of RAX mRNA. **A**: A map of the 3′-UTR of *RAX* mRNA was generated to indicate the putative binding sites, as predicted by the three bioinformatic algorithms. The seed region of this common miRNA binding site is highly conserved among mammals. **B**: The diagram shows the target sites for the microRNA-29b (*miR-29b*) paralogs in the 3′- untranslated region (3′-UTR) of PKR associates protein X (RAX) mRNA, including the RNA hybrid-free energy calculations and the theoretical miRNA-mRNA duplex pairing. The colon (:) represents the location of a G/U wobble base pairing.

The results of the computational analysis indicated that one of the *miR-29* targets is *RAX* mRNA. As shown in Figure 3A, there is a region of sequence in the 3′-UTR of *RAX* that is highly conserved across all mammals and has identical nucleotides from the second to the seventh base; this is known as the “seed” sequence. The seed sequence is considered to be the most critical sequence for selecting targets of miRNAs. We found that the three paralogs of *miR-29* (*miR-29a*, *miR-29b*, and *miR-29c*) have a complementary sequence to the seed sequence on *RAX* with minor divergences (Figure 3B), suggesting that the three paralogs potentially target *RAX* mRNA. The localization of the binding sites of these paralogs of *miR-29* to on the *RAX* 3′-UTR overlap, and there is only a single target site on *RAX* for each miRNA studied.

### Cellular localization of PKR associated protein X (RAX) protein in the retina

**Figure 4:**
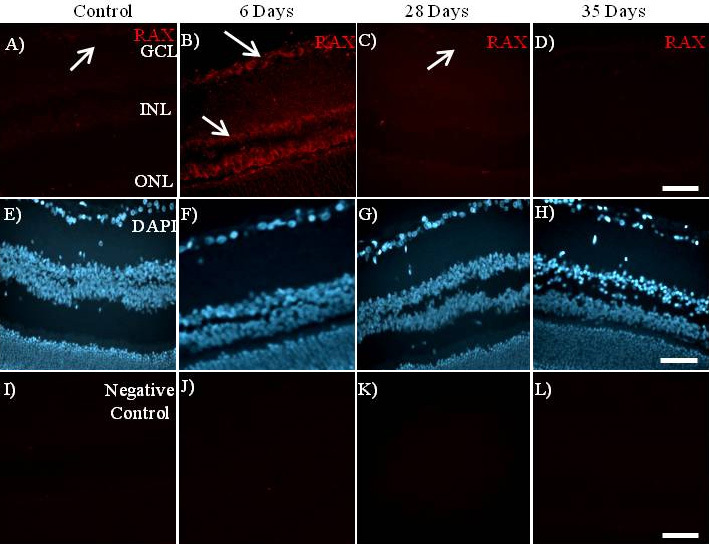
The cellular localization of PKR associated protein X (RAX) in the retinas of streptozotocin-induced diabetic rats by immunofluorescence. The retinas of diabetic rats show a strong cytoplasmic expression of RAX at 6 days (**B**) after STZ injection when compared to normal rats (**A**). The RAX expression in the retinas of diabetic animals at 28 days (**C**) and 35 days (**D**) after STZ injection was strongly reduced to the level of normal rats (**A**). The nuclei of cells (**E**, **F**, **G**, and **H**) were stained with the 4',6-diamidino-2-phenylindole (DAPI, blue) and the negative controls sections (**I**, **J**, **K**, and **L**) were incubated without the anti-RAX antibody. The negative control sections (incubated without the anti-RAX antibody) of the normal (**I**) and diabetic rat retinas at 6, 28, and 35 days are **J**, **K**, and **L**, respectively. Arrows indicate that RAX protein was expressed in the ganglion cell layer and the inner nuclear layer of the retina. The original magnification was 400×. The scale bar represents 50 μm. Abbreviations are as follows: GCL represents ganglion cell layer; INL represents inner nuclear layer; ONL represents outer nuclear layer; SRS represents subretinal space. The results shown in this figure are representative of three replicates.

The analysis by immunofluorescence demonstrated that RAX protein is expressed in the same specific retinal cell types as that observed for *miR-29b* expression (Figure 1B and Figure 4B). The RAX-positive cells are the same in the retinas of normal and diabetic rats (Figure 4A,B); however, there is strong cytoplasmic expression of RAX at 6 days after STZ injection when compared to normal rats. In the retinas of diabetic animals at 28 and 35 days after STZ injection, RAX expression was strongly reduced to the level of normal rats (Figure 4A,C,D).

### Expression of PKR associated protein X (RAX) mRNA and protein in the retina

**Figure 5:**
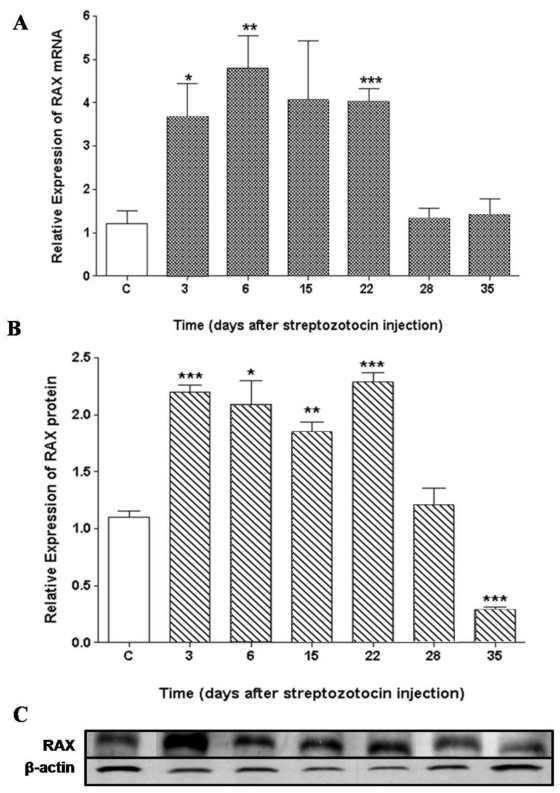
PKR associated protein X (*RAX*) expression in the retinas of rats after treatment with streptozotocin. **A**: *RAX* mRNA expression was evaluated by qRT–PCR and was normalized to *ACTB*. **B**: RAX protein expression was evaluated by western blot using anti-RAX antibody. Rats were used in groups of three. Data are means±standard error of the mean from three independent experiments, and each sample was run in six replicates. **C**: The immunoblot is representative of one typical experiment. For both the mRNA and the protein levels of RAX, the paired Student *t* test was performed to ascertain whether there is statistical significance between the retinas of diabetic rats and nondiabetic rats. The asterisks (*, ** and ***) indicate statistical significance (p<0.05, p<0.005 and p<0.0005, respectively) between control and experimental groups.

PKR associated protein X (*RAX*) mRNA expression in the retinal cells was significantly higher (>3.5-fold) than that of the control group at 3 (p=0.04), 6 (p=0.004), and 22 (p=0.001) days after injection of STZ, and the expression levels returned to that of the control by 28 days (Figure 5A). The expression of RAX protein was increased (>1.5 fold) at 3, 6, 15, and 22 days (p=0.001, p=0.01, p=0.002, p<0.001, respectively) and decreased to a level below that of the control group at 35 days after STZ injection (p<0.001; Figure 5B).

### Analysis of the microR-29b target by luciferase reporter assay

**Figure 6:**
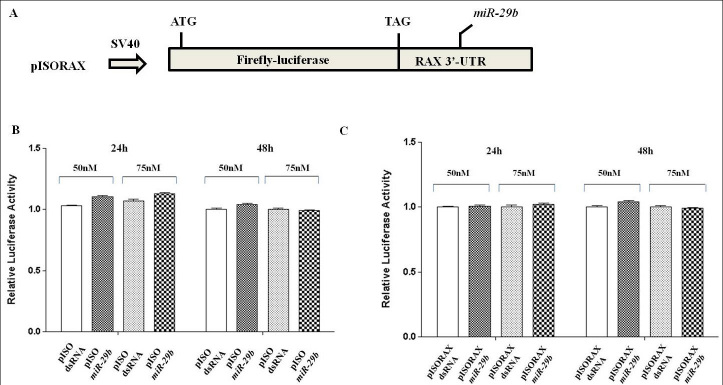
Luciferase reporter assay. **A**: The firefly luciferase reporter construct (pISORAX plasmid) contains a partial-length 3′-UTR of RAX mRNA in the 5′ to 3′ orientation with respect to the firefly-luc open reading frame with the firefly luciferase start and stop codons and the potential *miR-29* target site. The pISORAX plasmid contains the partial-length 3′-UTR of RAX mRNA in the 5′ to 3′ orientation with respect to the firefly-luc open reading frame. The firefly luciferase start and stop codons and the potential *miR-29* target site are also shown. HEK293 cells were co-transfected with pISO (**B**) or pISORAX (**C**) and pRL-TK together with a synthetic *miR-29b* mimic or dsRNA as a negative control. The indicated concentrations refer to the quantity of *miR-29b* mimic or dsRNA used for the transfection. The luciferase activity of firefly-luc and *Renilla*-luc were measured at 24 h and 48 h after transfection. The firefly-luc activity was normalized to *Renilla*-luc expression. Data are means±standard error of the mean from three independent experiments, each containing three replicates.

To investigate whether *miR-29b* directly regulates *RAX* expression, we used a luciferase reporter assay. HEK293 cells were co-transfected with the plasmids (pISO-RAX or pISO empty) and a synthetic mimic of *miR-29b* or dsRNA (negative control). The levels of luciferase activity were measured at 24 and 48 h after transfection to determine the repressive effects of this miRNA. The dual-luciferase activity assay revealed that there was no difference in luciferase activity between the cells transfected with the negative control and the *miR-29b* oligonucleotides (Figure 6B,C).

### The effects of overexpression of microR-29b on PKR associated protein X (*RAX*) mRNA and protein levels

**Figure 7:**
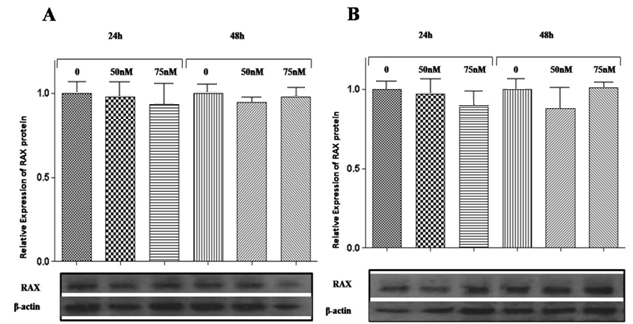
PKR associated protein X (RAX) expression in the presence of a microRNA-29b mimic. The expression of RAX protein was evaluated by western blot analysis. **A**: The expression of RAX protein in CJ4 cells transfected with a *miR-29b* mimic at a concentration of 0, 50, or 75 nM after 24 and 48 h of transfection. **B**: The expression of RAX protein in CJ4 cells transfected with dsRNA (negative control) at a concentration of 0, 50, or 75 nM, 24 and 48 h after transfection. β-actin was used as a loading control. Rats were used in groups of three. Data are means±standard error of the mean from three independent experiments, and each sample was run in six replicates. The immunoblot is representative of one typical experiment.

**Figure 8:**
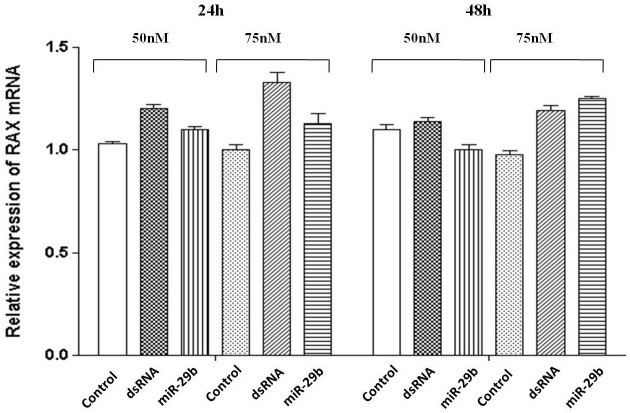
PKR associated protein X (RAX) mRNA expression in the presence of a *microRNA-29b* mimic. The expression of *RAX* mRNA was evaluated by qRT–PCR in CJ4 cells transfected with an *miR-29b* mimic or dsRNA (negative control) at a concentration of 50 or 75 nM, 24 h (**A**) and 48 h (**B**) after transfection. Nontransfected CJ4 cells were used as a control. Data are means±standard error of the mean from three independent experiments, each containing three replicates.

When a specific mRNA is a target of an miRNA, a change in the concentration of the miRNA in the cell causes a change in the amount of the mRNA and/or protein of the target in the cell. CJ4 cells were transfected with a synthetic mimic of *miR-29b* or dsRNA (negative control) in concentrations of 0, 50, and 75 nM, and the expression of RAX protein was evaluated at 24 and 48 h after transfection. The results revealed no significant changes in the protein expression of RAX (Figure 7A,B). Although there is little information [27] in the literature showing the regulation of gene expression by miRNAs in mammalian cells through the degradation of the mRNA target, we decided to investigate this possibility in our system. With this objective, CJ4 cells were transfected in the same conditions above and the total RNA was extracted from the transfected cells at 24 and 48 h. The analysis by qRT–PCR revealed that there was no significant change in the expression level of *RAX* mRNA (Figure 8).

## Discussion

It was previously demonstrated that RGCs undergo apoptosis in STZ-induced diabetic rats [9,10]. Recent studies have indicated that *miR-29b* is involved in apoptosis [12,13]; therefore, the first step was to investigate the cellular localization of *miR-29b* in the retinas of normal and diabetic animals. We observed, by in situ hybridization, that *miR-29b* is highly expressed in the neurons of the RGC of diabetic rats. The analysis of the expression of *miR-29b* in the retina by qRT–PCR showed that *miR-29b* is upregulated at 28 and 35 days after STZ injection. Considering the localization results from in situ hybridization, it is reasonable to assume that the *miR-29b* expression detected by qRT–PCR reflects its expression in RGCs and the cells of the INL of the rat retina.

The next step was to examine the potential targets of *miR-29b,* as it is known that several miRNAs regulate an overlapping set of target genes [12,13,28]. The computational prediction programs suggested that *RAX* is a target of the *miR-29* family (*miR-29a*, *miR-29b,* and *miR-29c*), and *miR-29b* showed the highest score of prediction. It was reported that RAX, an activator of PKR, and its human ortholog PACT are 98% identical and ubiquitously expressed in mammalian cells [17,29]. Recently, several studies have shown that *RAX* is upregulated during cellular stress [17,30-32]. Moreover, it was found that PKR plays a role in the regulation of important cellular processes, including the apoptosis of RGCs and neurons in patients with Parkinson, Alzheimer, and Huntington’s diseases [33-37]. Interestingly, PKR is a stress-responsive kinase, and it has been described that apoptosis in mammalian cells occurs in response to stress [15]. Thus, it was found that PKR has a significant role in ER stress-dependent apoptosis through the eIF-2α/ATF4/CHOP signaling pathway [38]. These authors also observed that ER stress does not induce PKR expression but rather activates the pre-existing PKR via the induction of the PKR-activating protein PACT. More recently, it was suggested that hyperglycemia induces ER stress in the tubular cells of the kidney in patients with established diabetic nephropathy [39]. In addition, the intracellular accumulation of glucosamine observed in the cells of diabetic animals may promote the misfolding of proteins in the ER lumen and consequently ER stress [40]. On the basis of these observations, we speculated that *RAX* is upregulated in the retinal neurons of STZ-induced diabetic rats. To test this hypothesis, we investigated the cellular localization and the expression of RAX protein in the retina of STZ-induced diabetic rats.

Clearly, the miRNA and its mRNA target must be expressed in the same cells, and they are expected to have an inverse correlation of expression. The analysis of the cellular localization of RAX by immunofluorescence indicated that RAX is also strongly expressed in the neurons of the ganglion cell layer of the retinas of diabetic animals. These findings suggest that, if the *miR-29b* target site in the 3′-UTR of *RAX* mRNA is accessible in vivo, the expression of *RAX* may be negatively regulated by *miR-29b*. Interestingly, the upregulation of *miR-29b* at 28 and 35 days after the injection of STZ is accompanied by the downregulation of RAX protein. This suggests that *RAX* expression is negatively regulated by *miR-29b* and may represent a mechanism of protection of retinal neurons against apoptosis, which occurs around 35 days after STZ-induced diabetes [41]. Recently it was found that *miR-29b* has a protective effect against the deposition of extracellular matrix in the trabecular meshwork cells when induced by chronic oxidative stress, as observed in glaucoma [15].

To gain more insight into the role played by *miR-29b* in the retina during the early stage of STZ-induced diabetes, we investigated whether *RAX* is a direct target of *miR-29b*. The luciferase assay and the overexpression of *miR-29b* did not validate RAX as a direct target of *miR-29b*. Thus, it is possible that in the retina of diabetic rats *miR-29b* is acting on another target rather than *RAX* or this PKR activator is an indirect target of *miR-29b*. Not all the targets predicted by computational programs are experimentally validated [42]. In addition, an inverse correlation of expression of an miRNA and its putative target may suggest that miRNAs function indirectly. Moreover, it has been observed that miRNAs act as indirect regulators of gene expression through the regulation of transcription factors [43]. It is estimated that approximately a quarter of the putative targets of miRNAs are transcription factors, suggesting that changes in the expression of an miRNA may modulate the activity of specific transcription factors and alter gene regulatory circuits in physiologic and pathological conditions [44].

Recently, it was reported that *miR-29b* indirectly regulates DNA methyltransferase 1 (DNMT1) by targeting the transcription factor Sp1 which binds to the GC boxes in the promoter of the DNMT1 gene in mice and humans [45-47]. Interestingly, the promoter of PACT and RAX contains GC boxes, indicating that the expression of these PKR activators is regulated by Sp1 [48]. These findings prompted us to perform in silico analysis to search binding sites for *miR-29b* in the 3′-UTR of rat *Sp1* mRNA. Our results revealed that the binding sites for *miR-29b* are conserved in human and rat, suggesting that *miR-29b* may also regulate *Sp1* expression in rat (data not shown). It should be emphasized that Sp1 is involved in the transcription of several genes regulated by *miR-29b* [28].

**Figure 9:**
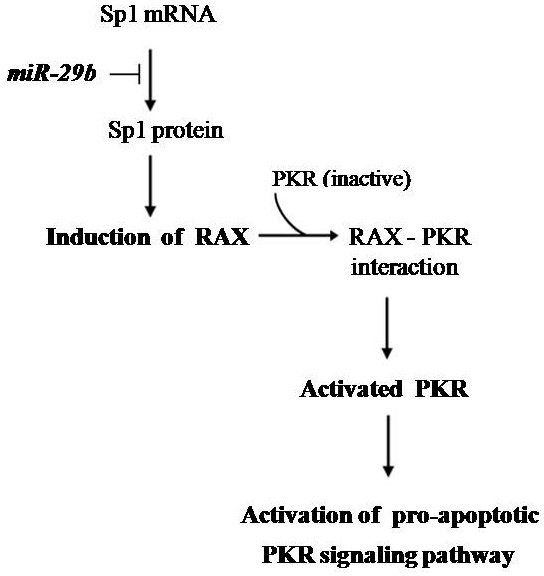
Overview of the proposed mechanism by which microR-29b indirectly regulates the expression of PKR associated protein X (RAX) in the retinal neurons of the rat. According to our hypothesis, the upregulation of *miR-29b* in retinal neurons of diabetic rats may result in down-regulation of RAX which decreases the activated PKR level with subsequent reduction of the activity of the pro-apoptotic PKR signaling pathway. Abbreviations: Sp1 represents transcription factor Specificity protein 1; miR-29b represents microRNA-29b; RAX represents PKR associated protein X; PKR represents RNA-dependent protein kinase.

Based on the results of this work and that from the literature, it is reasonable to speculate that *RAX* expression is indirectly regulated by *miR-29b* in the retinal neurons of the rat (Figure 9). Thus, the downregulation of RAX protein that was observed in the rat retina at 28 and 35 days after injection of STZ could be explained by the inhibition of *Sp1* expression due to the upregulation of *miR-29b*. Moreover, the downregulation of *RAX* could result in a decrease of the activated PKR level with subsequent reduction of the activity of the pro-apoptotic PKR signaling pathway. It is important to note that the apoptosis of the retinal neurons in our experimental model of diabetes occurs around 35 days after STZ injection [10]. Therefore, it is possible that the upregulation of *miR-29b* at 28 and 35 days may represent part of an adaptive response to protect the retinal neurons against apoptosis by the PKR signaling pathway in diabetic rats. However, additional experiments are required to confirm our working hypothesis, such as the validation of rat Sp1as a direct target of *miR-29b* and the evaluation of the expression of *Sp1* and phosphorylated (activated) Sp1 in the retinas of normal and diabetic rats. The levels of activated Sp1 should be examined to determine if the phosphorylation of pre-existing Sp1 can explain the apparently *miR-29b*-independent induction of *RAX* observed at 6 days after injection of STZ.

To our knowledge, this is the first evidence for miRNA involvement in the apoptosis of retinal neurons. It should be emphasized that the apoptosis of retinal neurons plays a crucial role in the pathogenesis of DR [9,10]. Moreover, our results provide a new focus for future studies and may contribute to the development of new strategies for the treatment of DR, such as the intravitreal injection of *miR-29b.* Recent studies suggest a potentially therapeutic use of miRNAs and small interfering RNAs for human diseases. This represents a promising and exciting area of biomedical research.

## References

[1] Bartel DP (2004). MicroRNAs: genomics, biogenesis, mechanism, and function.. Cell.

[2] Lewis BP, Burge CB, Bartel DP (2005). Conserved seed pairing, often flanked by adenosines, indicates that thousands of human genes are microRNA targets.. Cell.

[3] Wang Y, Baskerville S, Shenoy A, Babiarz JE, Baehner L, Blelloch R (2008). Embryonic stem cell-specific microRNAs regulate the G1-S transition and promote rapid proliferation.. Nat Genet.

[4] Chen CZ (2004). Ling Li, Harvey FL, David PB. MicroRNAs Modulate Hematopoietic Lineage Differentiation.. Science.

[5] Esau C, Kang X, Peralta E, Hanson E, Marcusson EG, Ravichandran LV, Sun Y, Koo S, Perera RJ, Jain R, Dean NM, Freier SM, Bennett CF, Lollo B, Griffey R (2004). MicroRNA-143 regulates adipocyte differentiation.. J Biol Chem.

[6] Loscher CJ, Hokamp K, Wilson JH, Li T, Humphries P, Farrar GJ, Palfi A (2008). A common microRNA signature in mouse models of retinal degeneration.. Exp Eye Res.

[7] Xu S, Witmer DP, Lumayag S, Kovacs B, Valle D (2007). MicroRNA (miRNA) Transcriptome of Mouse Retina and Identification of a Sensory Organ-specific miRNA Cluster.. J Biol Chem.

[8] Fong DS, Aiello L, Gardner TW, King GL, Blankenship G, Cavallerano JD, Ferris FL, Klein R, American Diabetes Association (2003). Diabetic retinopathy.. Diabetes Care.

[9] Bresnick GH (1986). Diabetic retinopathy viewed as a neurosensory disorder.. Arch Ophthalmol.

[10] Barber AJ, Lieth E, Khin SA, Antonetti DA, Buchanan AG, Gardner TW (1998). Neural apoptosis in the retina during experimental and human diabetes: early onset and effect of insulin.. J Clin Invest.

[11] Asnaghi V, Gerhardinger C, Hoehn T, Adeboje A, Lorenzi M (2003). A role for the polyol pathway in the early neuroretinal apoptosis and glial changes induced by diabetes in the rat.. Diabetes.

[12] Mott JL, Kobayashi S, Bronk SF, Gores GJ (2007). *miR-29* Regulates Mcl-1 Protein Expression and Apoptosis.. Oncogene.

[13] Park SY, Lee JHH, Ha M, Nam JWW, Kim VNN (2009). *miR-29* activate p53 by targeting p85alpha and cdc42.. Nat Struct Mol Biol.

[14] Arora A, McKay GJ, Simpson DAC (2007). Prediction and Verification of miRNA Expression in Human and Rat Retinas.. Invest Ophthalmol Vis Sci.

[15] Lee ES, Yoon C, Kim Y, Bae Y (2007). The double-strand RNA-dependent protein kinase PKR plays a significant role in a sustained ER stress-induced apoptosis.. FEBS Lett.

[16] Li B, Wang HS, Li GG (2010). Zhao M j, Zhao M H. The role of endoplasmic reticulum stress in the early stage of diabetic retinopathy.. Acta Diabetol.

[17] Ito T, Yang M, May WS (1999). RAX, a cellular activator for double-stranded RNA-dependent protein kinase during stress signaling.. J Biol Chem.

[18] Wei M, Leslie O, Maree TS, Fraser BR, Katrina S, Andrew JH, Darryl B, Lindsay B (2003). The Streptozotocin-Diabetic Rat as a Model of the Chronic Complications of Human Diabetes.. Heart Lung Circ.

[19] Pepato MT, Migliorini RH, Goldberg AL, Kettelhut IC (1996). Role of different proteolytic pathways in degradation of muscle protein from streptozotocin-diabetic rats.. Am J Physiol Endocrinol Metab.

[20] Kloosterman WP, Plasterk RH (2006). The diverse functions of microRNAs in animal development and disease.. Dev Cell.

[21] Ryan DG, Oliveira-Fernandes M, Lavker RM (2006). MicroRNAs of the mammalian eye display distinct and overlapping tissue specificity.. Mol Vis.

[22] Pfaffl MW (2001). A new mathematical model for relative quantification in real-time RT-PCR.. Nucleic Acids Res.

[23] Zanette DL, Rivadavia F, Molfetta GA, Barbuzano FG, Proto-Siqueira R, Falcão RP, Zago MA, Silva-Jr WA (2007). miRNA expression profiles in chronic lymphocytic and acute lymphocytic leukemia.. Braz J Med Biol Res.

[24] Lowry OH, Rosebrough NJ, Farr AL, Randall RJ (1951). Protein measurement with the folin phenol reagent.. J Biol Chem.

[25] Chen CZ, Li L, Lodish HF, Bartel DP (2004). MicroRNAs modulate hematopoietic lineage differentiation.. Science.

[26] Naguibneva I, Ameyar-Zazoua M, Polesskaya A, Ait-Si-Ali S, Groisman R, Souidi M, Cuvellier S, Harel-Bellan A (2006). The microRNA *miR-181* targets the homeobox protein Hox-A11 during mammalian myoblast differentiation.. Nat Cell Biol.

[27] Valencia-Sanchez MA, Liu J, Hannon GJ, Parker R (2006). Control of translation and miRNA degradation by miRNAs and siRNAs.. Genes Dev.

[28] Luna C, Li G, Qiu J, Epstein DL, Gonzalez P (2009). Role of *miR-29b* on the regulation of the extracellular matrix in human trabecular meshwork cells under chronic oxidative stress. Mol Vis.

[29] Patel RC, Sen GC (1998). PACT, a protein activator of the interferon-induced protein kinase, PKR.. EMBO J.

[30] Bennett RL, Blalock WL, Abtahi DM, Pan Y, Moyer SA, May WS (2006). RAX, the PKR activator, sensitizes cells to inflammatory cytokines, serum withdrawal, chemotherapy, and viral infection.. Blood.

[31] Bennett RL, Blalock WL, May WS (2004). Serine 18 Phosphorylation of RAX, the PKR activator, is required for PKR activation and consequent translation inhibition.. J Biol Chem.

[32] Patel CV, Handy I, Goldsmith T, Patel RC (2000). PACT, a stress-modulated cellular activator of interferon-induced double-stranded RNA-activated protein kinase, PKR.. J Biol Chem.

[33] Bando Y, Onuki R, Katayama T, Manabe T, Kudo T, Taira K, Tohyama M (2005). Double-strand RNA dependent protein kinase (PKR) is involved in the extrastriatal degeneration in Parkinson's disease and Huntington's disease.. Neurochem Int.

[34] Page G, Rioux Bilan A, Ingrand S, Lafay-Chebassier C, Pain S, Perault Pochat MC, Bouras C, Bayer T, Hugon J (2006). Activated double-stranded RNA-dependent protein kinase and neuronal death in models of Alzheimer's disease.. Neuroscience.

[35] Watanabe MAE, Souza LR, Murad JM, De Lucca FL (2005). Activation of the RNA-dependent protein kinase of lymphocytes by regulatory RNAs: implications for immunomodulation in HIV infection.. Curr HIV Res.

[36] Delgado André N, De Lucca FL (2008). Non-coding transcript in T cells (NTT): antisense transcript activates PKR and NF-kappaB in human lymphocytes.. Blood Cells Mol Dis.

[37] Murad JM, de Souza LR, De Lucca FL (2006). PKR activation by a non-coding RNA expressed in lymphocytes of mice bearing B16 melanoma.. Blood Cells Mol Dis.

[38] Lee ES, Yoon CH, Kim YS, Bae YS (2007). The double-strand RNA-dependent protein kinase PKR plays a significant role in a sustained ER stress-induced apoptosis.. FEBS Lett.

[39] Lindenmeyer MT, Rastaldi MP, Ikehata M, Neusser MA, Kretzler M, Cohen CD, Schlöndorff D (2008). Proteinuria and hyperglycemia induce endoplasmic reticulum stress.. J Am Soc Nephrol.

[40] Werstuck GH, Khan MI, Femia G, Kim AJ, Tedesco V, Trigatti B, Shi Y (2006). Glucosamine-induced endoplasmic reticulum dysfunction is associated with accelerated atherosclerosis in a hyperglycemic mouse model.. Diabetes.

[41] Barber AJ, Lieth E, Khin SA, Antonetti DA, Buchanan AG, Gardner TW (1998). Neural apoptosis in the retina during experimental and human diabetes: early onset and effect of insulin.. J Clin Invest.

[42] Sethupathy P, Megraw M, Hatzigeorgiou AG (2006). A guide through present computational approaches for the identification of mammalian microRNA targets.. Nat Methods.

[43] Xiao C, Calado DP, Galler G, Thai TH, Patterson HC, Wang J, Rajewsky N, Bender TP, Rajewsky K (2007). MiR-150 controls B cell differentiation by targeting the transcription factor c-Myb.. Cell.

[44] Saba R, Goodman CD, Huzarewich RL, Robertson C, Booth SA (2008). A miRNA signature of prion induced neurodegeneration.. PLoS ONE.

[45] Kishikawa S, Murata T, Kimura H, Shiota K, Yokoyama KK (2002). Regulation of transcription of the Dnmt1 gene by Sp1 and Sp3 zinc finger proteins.. Eur J Biochem.

[46] Liu S, Liu Z, Xie Z, Pang J, Yu J, Lehmann E, Huynh L, Vukosavljevic T, Takeki M, Klisovic RB, Baiocchi RA, Blum W, Porcu P, Garzon R, Byrd JC, Perrotti D, Caligiuri MA, Chan KK, Wu LC, Marcucci G (2008). Bortezomib induces DNA hypomethylation and silenced gene transcription by interfering with Sp1/NF-kappaB-dependent DNA methyltransferase activity in acute myeloid leukemia.. Blood.

[47] Garzon R, Liu S, Fabbri M, Liu Z, Heaphy CEA, Callegari E, Schwind S, Pang J, Yu J, Muthusamy N, Havelange V, Volinia S, Blum W, Rush LJ, Perrotti D, Andreeff M, Bloomfield CD, Byrd JC, Chan K, Wu LC, Croce CM, Marcucci G (2009). MicroRNA-29b induces global DNA hypomethylation and tumor suppressor gene re-expression in acute myeloid leukemia by targeting directly DNMT3A and 3B and indirectly DNMT1.. Blood.

[48] Fasciano S, Kaufman A, Patel RC (2007). Expression of PACT is regulated by Sp1 transcription factor.. Gene.

